# Assessment of Sensory Processing Characteristics in Children between 3 and 11 Years Old: A Systematic Review

**DOI:** 10.3389/fped.2017.00057

**Published:** 2017-03-30

**Authors:** Sara Jorquera-Cabrera, Dulce Romero-Ayuso, Gemma Rodriguez-Gil, José-Matías Triviño-Juárez

**Affiliations:** ^1^Facultad de Terapia Ocupacional, Logopedia y Enfermería, Psychology, Universidad de Castilla-La Mancha, Talavera de la Reina, Spain; ^2^Centro de Terapia Infantil AYTONA, Madrid, Spain; ^3^West Health District, Primary Care Center Francia, Madrid Health Service, Madrid, Spain

**Keywords:** assessment, children, sensory integration, sensorial modulation, sensory processing

## Abstract

The assessment of sensory perception, discrimination, integration, modulation, praxis, and other motor skills, such as posture, balance, and bilateral motor coordination, is necessary to identify the sensory and motor factors influencing the development of personal autonomy. The aim of this work is to study the assessment tools currently available for identifying different patterns of sensory processing. There are 15 tests available that have psychometric properties, primarily for the US population. Nine of them apply to children in preschool and up to grade 12. The assessment of sensory processing is a process that includes the use of standardized tests, administration of caregiver questionnaires, and clinical observations. The review of different studies using PRISMA criteria or Osteba Critical Appraisal Cards reveals that the most commonly used tools are the Sensory Integration and Praxis Test, the Sensory Processing Measure, and the Sensory Profile.

Sensory processing is a broad term that generally refers to the handling of sensory information by neural systems, including the functions of receptor organs and the peripheral and central nervous systems. According to Dunn, sensory processing is a complex endeavor. Sensory input from the environment and from the body itself provides information to the brain (1). The brain organizes, integrates, synthesizes, and uses this information to understand experiences and organize appropriate responses. The processing of information allows individuals to respond automatically, efficiently, and comfortably to the specific sensory inputs received (2, 3). The neurobiological process comprises a series of five stages, registration, modulation, discrimination, integration, and praxis (4), and is central to cognitive processes such attention, visual perception, memory, and planned action (5).

Ayres paid special attention to the relationship between motor responses, sensory input, and normal sensorimotor development. She defined sensory integration (SI) as the ability to organize sensory information to make an adaptive response (6). Recently, some authors have suggested that SI should be referred to as multisensory integration (7). Behaviors associated with sensory processing are not necessarily symptoms or abnormalities; these are differences and often abilities, such as enhanced perception (8). For this reason, some authors prefer to use sensory features (9).

Ayres focused particularly on the identification of different patterns of dysfunction in sensorimotor development and their impact on learning and on the description of adaptive behaviors observed in children with motor clumsiness or learning disabilities of unknown origin (10–12). Sensory processing disorder (SPD) “affects the way the brain interprets the information that comes in and the response that follows, causing emotional, motor, and other reactions that are inappropriate and extreme” (13) (p. 331). Parham and Mailloux (14) outlined five functional impairments associated with SPD. These include decreased social participation and occupational engagement; decreased length, frequency, or complexity of adaptive responses (successful response to an environmental challenge); impaired self-confidence and/or self-esteem; poor daily life skills and reduced family life; and diminished fine-, gross-, and sensory–motor skill development. SPD can negatively affect development and functional abilities in behavior, emotional, motor, and cognitive domains (15). Consequently, it is important to detect differences early with appropriate sensory processing assessment tools.

Children diagnosed with various conditions, including autism spectrum disorder, Asperger syndrome, attention-deficit hyperactivity disorder, sensory-modulation disorder (SMD), and developmental coordination disorder, are prone to experience differences in their sensory processing patterns when compared to expected patterns (15–20). The Diagnostic Classification of Mental Health and Developmental Disorders of Infancy and Early Childhood (Zero to Three, 2005), which is the most commonly used diagnostic classification for early childhood includes a classification of “sensory processing regulation difficulties.” Furthermore, the classification proposed by *The Interdisciplinary Council on Development and Learning Disorders Diagnostic Manual for Infants and young Children* (ICDL-DMIC) also recognizes Regulatory-Sensory Processing Disorders (21). Moreover, the DSM-5 includes sensory perception disorders as a new diagnostic criterion for autism spectrum disorder (22).

There are different taxonomies to characterize differences in sensory processing. Recently, several authors have referred to other terms of these processes, especially in relation to possible modulation disorders such as hypo-reactivity, hypo-sensitivity to certain clinical observations and the presence of tactile defensiveness, enhanced perception, etc (7). However, the most commonly used and accepted taxonomy is that proposed by Miller et al. (23), who suggest that there are three main patterns: SMD, sensory-based motor disorder, and sensory discrimination disorder.

Sensory-modulation disorder refers to the difficulty in regulating and organizing the degree, intensity, and nature of a response to sensory stimuli through graded and adaptive behavior. People with SMD are able to sustain attention, filter sensations, and remain at the appropriate level of alertness. Modulation disorder presents three characteristic patterns: (1) “*Sensory over-responsivity*,” also known as sensory sensitivity or sensory avoidance, is characterized by intense, negative responses to typical daily life experiences, affecting alertness, attention, social interaction and the level of activity, and self-care. Symptoms include avoidance, anxiety, and hypersensitivity, e.g., tactile defensiveness or gravitational insecurity (24–26). (2) “*Sensory under-responsivity*” also termed “low registration” is characterized by delayed or reduced responses to daily sensory events, affecting the level of alertness, attention, posture, and movement, motor coordination, and social interaction (27). Sensory under-responsivity usually co-occurs with postural disorder. (3) “S*ensory seeking/craving”* is characterized by an insatiable drive for enhanced sensory experiences (28). Children with sensory seeking crave intense sensory input in different settings, exhibit strong sensory preferences, demonstrate socially inappropriate behaviors, and have little awareness of danger as well as difficulty in completing tasks. They also exhibit reduced inhibitory control and behavioral disorganization (26).

Various studies have analyzed the etiology of SI disorders, identifying a genetic factor in sensory over-responsivity (29). Hypersensitive persons are considered to have a low neurological threshold and easily notice sensory input, meaning they are frequently distracted by movement, sounds, textures, or smells not perceived by others (30). In contrast, hyposensitive persons present low registration; they do not notice everyday sensory events. For example, they may not notice when someone comes into a room or when they have food or dirt on their face and hands (31).

Sensory-based motor disorders occur when persons have inappropriate body posture or voluntary movement and who exhibit deficits in motor planning, praxis, sequencing, fluidity, and control of movement as a result of sensory difficulties (32). Two subtypes exist, both of which are influenced by impaired discrimination and perception of sensations: (1) *postural dysfunction* describes a difficulty in exerting postural control during movement or resting in response to the demands of the environment or a motor task. Postural control involves interactions between the vestibular, proprioceptive, and visual system, providing a stable basis for coordinating movements of the head, eyes, trunk, and limbs, which are essential to dynamic and static movement. Postural control difficulties appear when there is dysfunction in the previously mentioned systems, exhibiting deficits in movement control, reduced righting and balance reactions, limited weight transfer and trunk rotation capacity, poor balance between flexion and extension of body parts, and bilateral motor coordination difficulties, leading to ineffective performance of motor tasks. (2) *Dyspraxia* is the impaired ability to conceive of plan, sequence, and execute novel actions. Praxis refers to a capacity involving three processes: ideation, motor planning, and execution (33). Children with dyspraxia show difficulty moving their bodies in space and are more likely to have accidents. They experiences challenges related to ideation of movement, need more time, and practice to learn a new skill and demonstrate decreased ability to generalize skills to other motor tasks, such as in the execution of complex motor activities (34).

Sensory discrimination disorder refers to sensory processing patterns affecting interpretation of the quality of sensory input, especially temporal and spatial characteristics. Discrimination disorders can occur in one or more systems (i.e., vestibular, proprioceptive, and the five basic senses) (23). Discrimination difficulties affect the ability to identify similarities and differences between inputs. Children with this disorder may exhibit impaired motor planning and difficulties in praxis, as well as learning difficulties, low self-confidence, and poor body schema.

In recent years, a number of studies have implemented different assessment tools to examine SPDs (32, 35–38). These studies can be classified according to whether they use standardized tests, structured observations, or interviews with parents and teachers (39, 40). The aim of the assessment process is to determine the impact of sensory processing problems on children’s functionality and participation in daily life.

In their review of the literature, Koenig and Rudney conclude that difficulties in sensory processing affect elements of occupational performance: play and leisure, social participation, development of autonomy, basic and instrumental activities of daily living, and education (41). Eeles et al. report that SPDs may be the cause of learning and development difficulties found in some children (4).

Between 40 and 80% of children and 3 and 11% of adults with neurodevelopmental disabilities are estimated to have difficulties in sensory processing (42, 43). Between 60 and 95% of children with autism spectrum disorders have differences in sensory processing (31, 43–46). Between 2.8 and 6.5% of the typically developing population is also reported to have difficulties in sensory processing (29, 47). More specifically, 5% of children between 0 and 3 years of age exhibit sensory processing differences (15). Consequently, for early detection of these differences, it is essential to identify the most appropriate and precise tool for assessing sensory processing, to determine whether SI difficulties are a significant factor in a child’s behavior and to provide appropriate intervention (4).

There currently exists only one systematic review of diagnostic tests for SPDs in children between 0 and 3 years of age (4). Thus, it is especially interesting to conduct a systematic review of the assessment of SPDs in older children between 3 and 11 years of age. To the best of our knowledge, this is the first systematic review of sensory processing for this age group.

## Aim

The aim of this work is to conduct a review of the assessment tools currently available for determining different patterns of SPDs in children between 3 and 11 years of age.

## Methodology

### Search Strategy

Between October, 20, 2014, and January, 3, 2016, we conducted an exhaustive search of the literature to identify the instruments available for assessing sensory processing in children aged between 3 and 11. This search was conducted in the following databases: Web of Science, MEDLINE, SCOPUS, Trip database, OTSeeker, and Plinio. The search strategy included the terms MeSH (“Child” OR “children”) AND (“assessment” OR “evaluation”) AND (“sensory integration” OR “sensory processing” OR “occupational therapy”) and included articles published between 2004 and 2015 in both Spanish and English.

**Figure 1:**
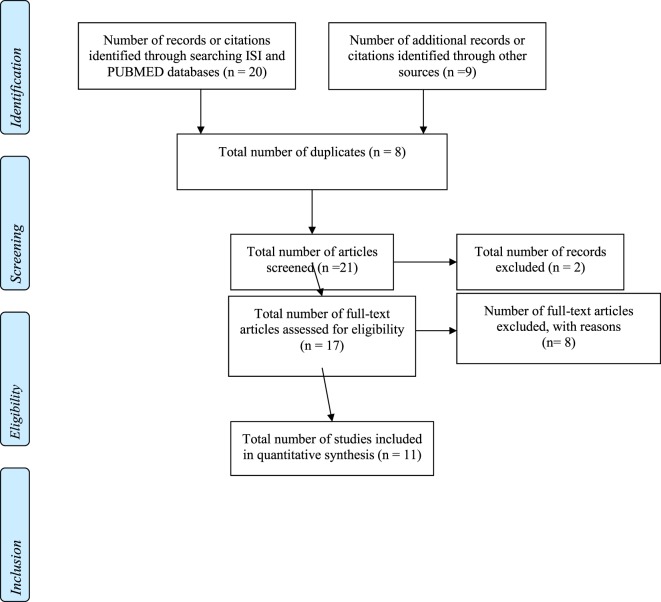
**PRISMA flow diagram**.

Two authors (Gemma Rodriguez-Gil and José-Matías Triviño-Juárez) reviewed the article titles and abstracts of the articles to determine whether they met the inclusion criteria. Two independent reviewers (Sara Jorquera-Cabrera and Dulce Romero-Ayuso) then reviewed the articles that were not selected to ensure they should be excluded. Any articles presenting doubts or inconsistencies were fully reviewed by the independent reviewers until a decision was finally reached on their inclusion or exclusion (see Figure 1 for a flow diagram adapted from the PRISMA methodology, see Table 1).

**Table 1:** **PRISMA checklist**.

Section/topic	Number	Item
Title	1	Assessments of sensory processing in infants: a systematic review
*Summary* Structured summary		Aim: To evaluate the psychometric properties and clinical use of assessments of sensory processing function during the first 2 years of life and to identify the most appropriate and precise assessment for measuring sensory processing Method: The literature was analyzed and the assessments used to measure sensory processing in early childhood were systematically selected and reviewed for clinical use, reliability, validity, and response capacity
	2	Results: 34 assessments were identified; three met the predefined inclusion criteria. All discriminatory assessments, the Sensory Assessment Scale and the Child Sensory Profile, are parent-reported questionnaires and can be administered up to the age of 3. The Test of Sensory Function in Infants is a performance-based assessment suitable for infants aged 4 to 18 months. Studies evaluating the psychometric properties of these three assessments differ in the properties evaluated and in reliability scores ranging from low to adequate Interpretation: Selection of the most appropriate and precise assessment for measuring sensory processing function in infancy depends on the specific components of sensory processing to be assessed, the child’s age and other sources of information regarding the child’s development
*Introduction* Rationale	3	The impact of early sensory processing capacities on learning and emotional development is unclear because of the difficulty of consistently defining concepts in the field and the lack of reliable and adequate assessments for detecting sensory dysfunctions in very young infants; this ambiguity arises because sensory integration (SI) theory is relatively new and is still being developed There is no consensus on a suitable tool for measuring sensory processing in early infancy. Despite the large number of appropriate motor assessments, most of these do not take sensory function into account. To advance scientific research and clinical practice in the field of sensory processing, the most appropriate and precise assessment tools need to be identified
Objectives	4	To evaluate the psychometric properties and clinical use of sensory processing assessments in the first 2 years of life and identify the most appropriate and precise assessment for measuring sensory processing
*Methods* Protocol and registration	5	There is no review protocol
Eligibility criteria	6	We conducted an exhaustive search of the assessments used to measure sensory processing in a large number of computerized databases, including Medline (1950 to April 2011), CINHL (1981 to April 2011), PsycINFO (1872 to April 2011), Embase (1980 to April 2011), and Web of Science (1900 to 2011)
Information sources	7	Medline, CINAHL, PsycINFO, Embase, and Web of Science
Search	8	The search strategy included MeSH terms (“Child behavior” OR “Sensation” OR “perception” OR “sensory processing” OR “psychomotor performance”) AND (“psychometrics” OR “outcome assessment” OR “questionnaire” OR “outcome and process assessment” OR “neuropsychological test” OR “reproducibility or results” OR “data interpretation, statistical” OR “observer variation”) AND (“infant” OR “premature infant” OR “low birth weight”)
Study selection	9	Inclusion criteria: Those used to assess sensory processing in babies, regardless of the gestational age at birth. Assessment of sensory processing with results at the age of 24 months or less, corrected for gestation. It was a criterion referring to child standards. Published in English. Most of the elements of assessment refer to sensory processing results. Considered multisensory modalities. Commercially available (test and manual). Exclusion criteria: Those used as detection tools (diagnostic tests, high sensitivity tests). Those focusing on infant behavior. Those measuring child–parent–therapist interaction. Language and communication tests. Social interaction tools. Cognitive and motor and mental development tests. Those mainly assessing motor capacity.
Data collection process	10	We evaluated the clinical use, reliability, validity, and response capacity of CanChild Critical Review Forms The characteristics of the tools were collected and documented, including the main objective of the assessment, assessment type, age range, and characteristics of the study sample
Data items	11	Infant behavior, sensation, perception, sensory processing, psychomotor development, psychometric, assessment results, questionnaire, assessment results and processes, neuropsychological test, reproduction results, interpretation of statistical data, observational variation, infancy, preterm baby, and low weight at birth
Risk of bias in individual studies	12	A verification list of the tools analyzed in the excluded articles was elaborated
Summary measures	13	No summary measures are specified
Synthesis of results	14	Data handling was subjective, in accordance with the established criteria
Risk of bias across studies	15	Risk of bias across studies was not evaluated
Additional analyses	16	The characteristics of the tools were collected and documented, including the main objective of the assessment, assessment type, age range, and characteristics of the study sample
*Results* Study selection	17	Only three assessment tools were selected from among the studies, which all used samples of children in the USA. Both the Child Sensory Profile and the Sensory Rating Scale are suitable for use with children from birth to 3 years, and the Test of Sensory Function in Infants is suitable for use with infants aged 4 to 18 months
Study characteristics	18	Data were collected on assessments meeting the predefined criteria. Accordingly, the following were found to be useful tools: the Sensory Rating Scale for Infants and Young Children: Development and reliability (49), the Child Sensory Profile (50), and the Test of Sensory Functions in Ref (51).
Risk of bias within studies	19	Risk of bias within studies was not measured
Results of individual studies	20	All the discriminatory assessments, the Sensory Assessment Scale, and the Child Sensory Profile are parent-reported questionnaires and can be administered up to the age of 3. The Test of Sensory Function in Infants is a performance-based assessment suitable for infants aged 4 to 18 months
Synthesis of results	21	We identified 34 assessments; three met the predefined inclusion criteria. The Sensory Rating Scale had a confidence interval of 61.1–75.8% and an internal consistency of 0.83; the Test of Sensory Function in Infants had a reliability interval of 56–68%. The Sensory Profile had an internal consistency of 0.83
Risk of bias across studies	22	Risk of bias within studies was not measured
Additional analyses	23	No additional analyses were conducted
*Discussion* Summary of evidence	24	Studies evaluating the psychometric properties of these three assessments differ in the properties evaluated and in reliability scores ranging from low to adequate
Limitations	25	One limitation is the difficulty of defining the constructs of sensory processing. Furthermore, the assessments measure slightly different components than those specified in the hypothesis
Conclusion	26	The selection of the most appropriate and precise assessment for measuring sensory processing in infants depends on the specific components of sensory processing that need to be assessed, the child’s age, and the information available on the child’s development from other sources (family, teachers)
*Funding* Funding	27	This study received funding from the Spanish National Health System and Council of Medical Research, the Cerebral Palsy Alliance, Daniel Family Grant, the Thyne Reid Foundation, the Myer Foundation and the Infrastructure Support Program of the Government of Victoria

### Inclusion Criteria

Inclusion of articles comprised two stages. In the first stage, we selected systematic reviews of sensory processing assessment, and in their absence, we included other articles on sensory processing tests and assessment tools. In the second stage, we selected the scales and tools presented in the studies. Tools meeting the following criteria were included: (1) usefulness in assessing sensory processing in children aged between 3 and 11 years; (2) accordance with the assumptions of, or be compatible with, SI theory (6, 12, 48); (3) demonstration of predictive, discriminatory, and/or evaluative value of sensory processing in children aged between 3 and 11 years; (4) published in English and/or Spanish; and (5) inclusion of items (more than 50%) that contain sensory processing results (visual, auditory, vestibular, proprioceptive and kinesthetic processing, tactile, olfactory, and taste processing) (see Table 2).

**Table 2:** Tools selected for the assessment of sensory processing in children aged 3–11 years.

Tool	Objective	Population	Applicability	Psychometric properties	Language in which the tests are available and the psychometric scores
Sensory processing measure (SPM) (52)	To assess sensory processing, praxis and social participation in different school environments and at home	SPM (5–12 years): home form, main classroom form, and school environments form. SPM-P (2–5 years) home and school forms	The scale is completed by teachers and caregivers who have known the child for more than a month	SPM was standardized with a sample of 1,051 typically developing children from the USA and Canada, aged 5–13 years. Also, 345 children receiving occupational therapy treatment was used to verify that SPM could help us differentiate typical children from those with clinical disorders. SPM-P was standardized with 651 typically developing children from the USA aged 2–5 years. Also, a sample of 242 children with occupational therapy treatment was used to verify that SPM-P let us differentiate typical children from those with clinical disorders. Good reliability and validity. Internal consistency (alpha coefficient) ≥0.75 for all scales and forms. SPM scales appropriately distinguished between a normative sample and a sample of clinic-referred children with sensory processing difficulties	Sensory Processing Measure-Hong Kong Chinese version (SPM-HKC). Cronbach’s alpha 0.80. ICC of the Main classroom form ranged from 0.82 to 0.98 and the ICC of the home form ranged from 0.70 to 0.95. Good discriminant validity. Moderate correlation between Sensory profile Chinese and SPM-HKC. It is available in Danish, Finnish, Norwegian, Swedish, and Arabic
Sensory profile (1,45,54)	Evaluates the type of responses and self-regulation strategies used by the child and the type of neurological threshold for different sensory stimuli	Different versions. It can be administered from 0 to 14 years. There is a second version (SP^2^) toddler, infant, child, short form (SSP) and school companion published in 2014	Scale is completed by teachers and parents	Sensory Profile was standardized with a sample of 1,037 children without disabilities, 32 children with autism and 61 with ADHD diagnosis. New version of Sensory profile, Sensory Profile 2 was standardized with a sample of 1,376 school-age children in the USA	Infant/toddler sensory profile. ICC > 0.90. Alpha coefficients varied from 0.40 to 0.74. Test–retest reliability = 0.81–0.90. India Sensory Profile Caregivers Questionnaire The interrater reliability (ICC = 0.87), test–retest reliability (ICC = 0.90), internal consistency (Cronbach’s α = 0.86), section total correlation, face, and content validity for the SPCQ were good. A threshold score of ≤481 in SPCQ was considered ideal as a cutoff score to identify cases of sensory processing dysfunction among Indian children. Sensory Profile for Chinese children with a good internal consistency (Cronbach’s α = 0.82). Test–retest reliability over a 2-week period (*r* = 0.93)
				ICC = 0.80–0.90 good test–retest reliability across quadrants, for factors ICC = 0.69–0.88 years ICC = 0.50–0.87 for scores in the composites of sensory processing, modulation, and behavioral and emotional responses. Internal consistency of the sections ranges from 0.70 to 0.90	
				SSP has a discriminant validity of >95% in identifying children with and without sensory processing differences	
Sensory Integration and Praxis Test (SIPT) (6)	To assess children’s sensory integration and praxis problems	Children aged from 4 to 8 years 11 months	Comprises 17 tests. Administered using visual demonstration and spoken instructions, except when assessing praxis. The lower the score, the greater the difficulty	Standardized with a sample of 1997 children in the USA. High psychometric properties	Available only in English, for USA population
DeGangi-Berk Test of Sensory Integration (TSI) (58)	Conducts a screening of SI dysfunction, with emphasis on the vestibular system. Assessment of postural and components and praxis. It is based on Assessment of Sensorimotor Integration in Preschool Children (DeGangi, 1979) (66)	Infant population aged 3–5 years	Comprises 36 items and assesses posture control, bilateral motor integration and reflex integration. The child completes various tests. Administration time is 30 min	Validity of domain and construct, stable inter-observer 0.84 and test–retest reliability. Standardized with a sample of 101 typical children and 38 developmental delayed children from US population	Available only in English
Touch Inventory for elementary school-aged children (TIE) (61)	Measures tactile defensiveness	Population 6–12 years. The criteria for administration are that the child needs to have the language competence of at least a 6-year-old, an IQ of at least 80 and no presence of physical disabilities (Royeen and Fortune 1990)	The 26-item Questionnaire. The response format for the TIE is 1 = no, 2 = a little, and 3 = a lot. Administered in 15 min, self-reported by child. The higher the score, the more the self-reported behaviors are indicative of tactile defensiveness	Standarized with a sample of 415 children from USA. Test –retest reliability (*r* = 0.91) with 1-week testing interval	Available only in English
Sensorimotor clinical observations (63–66)	Provides information on vestibular and proprioceptive functions. Mainly used to diagnose motor planning problems, vestibular, proprioceptive, proprioceptive-vestibular and motor deficits	From age 5	A tool that requires training and practice to be correctly administered and interpreted. Comprises 15 tests. Administration time between 30 and 40 min	High interrater reliability. Discriminative validity measured with a sample of children in Chile and the USA *p* < 0.01. Portuguese transcultural adaptation study (*N* = 201)	Available in English and Spanish
Comprehensive Observations of Proprioception (COP) (67)	The COP provides a reliable measure for detecting the origin of proprioceptive problems affecting children’s functional performance	Infant population from 2 years of age	Takes 15 min to administer and is designed for use in conjunction with sensorimotor observations or while observing a child’s free play	Sample size was 130 children. Intraclass correlation coefficient was 0.91. Validity found between results of COP and items from the SPM (body awareness) and the KIN (kinesthesia) and SWB (Standing and Walking Balance) tests from the SIPT	Available in English and Spanish
The Miller Assessment for Preschoolers (MAP) (68)	Assesses a child’s attention, social interaction, and sensory reactivity during the testing procedure provides a profile of sensory discrimination abilities, postural foundations, and praxis and screens for visual, perceptual, and language delays that could be affecting participation in the classroom	Test for children from 2 years, 9 months to 5 years, 8 months of age	Administration time 30 min. There are two forms: MAP Screening 27 Core test items (evaluation of attention, social interaction and sensory reactivity) and MAP Extended (behavior during testing, supplemental observations, developmental history: speech language, movement, draw a person), development history. 27 subtests in 5 domains: neurological foundations, motorcoordination, language, nonverbal cognition, and complex tasks (combined domains). The total MAP score is expressed in percentiles, and the cut-points are 0% to 5% (Red; likely problem, refer for evaluation), 6% to 25% (Yellow; possible problem, watch carefully and use clinical judgment about the need to refer for evaluation), and 26% to 99% (Green; unlikely to have problems, do not refer for assessment)	The MAP was standardized with a sample of 1,014 children. The MAP has excellent internal reliability (r = 0.79–0.82) and interrater reliability (r = 0.98). Test–retest reliability for total score is r = 0.81 Content validity for the MAP is supported in the literature as MAP total score correlates significantly with the WISC-R IQ scale (*r* = 0.50–0.45) and with the Woodcock-Johnson Math, Reading and Language subtests (*r* = 0.38–0.35)	Available in English, Japanese and Hebrew
Sensory Experiences Questionnaire Version 3.0 (SEQ-3.0) (7,69–72)	To obtain sensory characteristics and discriminate sensory patterns of hypo- and hyper-responsiveness among persons with autism, mental or developmental retardation	For 2–12 years	It is a 105-item parent report tool designed specifically to measure behavioral responses to naturally occurring sensory stimuli in the context of everyday situations in children with ASD. SEQ measures the frequency of sensory behaviors across four sensory response patterns (hypo-responsiveness, hyper-responsiveness, sensory interests, repetitions and seeking behaviors and enhanced perception), five modality categories (i.e., auditory, visual, tactile, gustatory/olfactory, vestibular/proprioceptive), and two contexts (i.e., social and non-social). The first 97 items measure the frequency using a 5-point Likert scale ranging from 1 (*never/almost never*) to 5 (*always/almost always*) with a higher score indicating more sensory symptoms. Caregiver takes approximately 15–20 min to complete the questionnaire	Has good internal consistency and test–retest reliability. Useful for assessing children with ASD. Psychometric study was conducted with 358 caregivers	Available only in English
The Sensory Processing Scales (SPS) Version 2.0 (28)	Evaluates sensory reactivity in seven domains: tactile (self-care and materials), auditory (sounds and places), visual, olfactory, gustatory, and vestibular-proprioception	4–19	Consists of a performance assessment of different activities and a caregiver-report inventory and a self-report form for adults. The results propose classifications of sensory over responsivity, sensory under responsivity, and sensory seeking. Administered in approximately 1 h. Consists of 27 subtests and 72 items across seven sensory domains (visual, auditory, tactile, vestibular, proprioceptive, gustatory, and olfactory). The activities are designed to resemble sensory experiences in daily life that generate atypical behavioral responses in children with sensory problems. Items within each subtest are scored to reflect the person’s responses at three time periods: (1) during the activity, (2) after the activity (<15 s), and (3) during the transition to the next activity	Standarized sample of 128 participants. Internal consistency is moderate to high, interrater reliability is moderate, and internal validity is statistically significant. Overall internal consistency yielded a 0.94, and domain reliabilities ranged from 0.79 to 0.93 (internal reliability >0.4) and discriminant validity (*p* < 0.01). The SPS Assessment appears to be a reliable and valid measure of sensory modulation (scale reliability >0.90; discrimination between group effect sizes >1.00). This scale has the potential to aid in differential diagnosis of sensory modulation issues	English
Test of Ideational Praxis (TIP) (73)	To examine a child’s ability to recognize and to interact with an and to evaluate ideation as a component of praxis	From 5 to 8 years. There is also a version for preschoolers, elaborated in 2014	A child is given a 24-inch long shoelace and is given the instruction, “Show me everything you can do with this string” and is then given 5 min to demonstrate the actions. A point is given for each action but the action must be demonstrated; description alone is not enough	Studies conducted in 2014 with 78 children aged 3, 4, and 5 years found, after 2 weeks, that the TIP had a high interrater reliability of 0.94 and a good test–retest reliability of 0.80	English
Motor Planning Maze Assessment (MPMA) (73)	To be used as a screening tool to identify deficits in motor performance and planning aspect of dyspraxia	Preschoolers from 3 to 5 years	Individually administered test consisting of three mazes. Application and correction takes 5 min	Has only been administered to 80 children in the USA. Interrater reliability was excellent on the total MPMA score [interclass correlation coefficient (0.96) and individual maze scores (0.90–0.98)]. The total MPMA score can distinguish developmental differences among preschoolers ages 3, 4, and 5 years. No differences were observed according to gender, race, or educational approach	English
Pediatric Clinical Test of Sensory Interaction for Balance (CTSIB) (74)	To evaluate a child’s ability to use visual, somatosensory, and vestibular input to maintain balance while standing	Over 6 years of age	The child must complete six tests, three on a stable surface and three on an unstable one. Some of the tests are performed with eyes closed and others with eyes open. In all conditions, the objective is to maintain balance for at least 30 s. Administration time is approximately 20 min	A tool with excellent interrater reliability (*r* = 0.88, range 0.60–1.00) for children between 4 and 9 years old. The sample data was 24 typical children. Validity of criteria: with proprioceptive disorders and the SOT. CTSIB shows which children have more modulation disorders and more reduced postural control than typically developing children for all visual stimuli (*p* < 0.05), except for somatosensory input with vision. There are only data from studies conducted in the USA. There is also a version for adults and older children	English
Classroom Sensory Environment Assessment (CSEA) (75)	Promote therapist–teacher collaboration to provide student support and classroom modification, for research on the impact of the sensory environment for children with ASD	Elementary school aged	161 items divided into sections by sensory type: vision (47), hearing (50), touch (20), movement (vestibular and proprioceptive; 25), smell (15), and taste (4). Items for the cafeteria, recess, and playground were included. The teachers rated items on the basis of a typical week. Teachers rated the frequency of occurrence of the sensory experience as no, never, or not applicable; rarely; occasionally; sometimes; and always. Next, if applicable, the teachers rated the intensity of the experience as weak, moderate, or strong	Classroom data (*N* = 152) were analyzed with counts, frequencies, means, and SDs. Reliability was examined with internal consistency ratings using Cronbach’s alpha. Skew and kurtosis were examined using the Kolmogorov–Smirnov test of normality and histogram. Interrater reliability was analyzed with intraclass correlation coefficients. The tool’s internal consistency is acceptable. Interrater reliability values did not reach acceptable levels in the pilot using the teacher–therapist rating pairs and total score. The ICC was −0.197. Cronbach’s alpha = 0.94. The current phase (Phase 4) included collection of descriptive data from a variety of elementary classrooms using the current version of the CSEA and an initial investigation of its internal consistency	English
Preschool Imitation and Praxis Scale (PIPS) (77,78)	The purpose of the Preschool Imitation and Praxis Scale (PIPS) is designated to be a reliable and valid multidimensional instrument to measure the accuracy of imitation performance of preschool children	1.5–4.9 years	40 PIPS items and 10 task categories of the PIPS: six gestural, three procedural and one facial. The positive and strong associations between the PIPS scale score and scores on mental, language and motor measures in children with autism spectrum disorders supported criterion-related validity	Psychometric study was conducted with 119 typically developing children. They demonstrated acceptable intra- and interrater reliability at the item level (0.45–1.00) and scale level. Exploratory factor analysis disclosed four dimensions on the scale: goal directed versus non-goal directed, procedural imitation, and single versus sequential bodily imitation. Internal consistency for the PIPS scale (*a* = 0.97) and subscales was high (a ranged from 0.79 to 0.96). In both samples, the PIPS scale score was strongly related to age (*r* = 0.78, respectively, *r* = 0.56). Significant relationships between the PIPS score and mental, language, motor ages in the ASD sample supported criterion-related validity (*r* ranged from 0.59 to 0.74)	English

### Exclusion Criteria

Tools meeting any of the following criteria were excluded: (1) fundamentally aimed at measuring mental or motor development; (2) aimed mainly at measuring a child’s motor ability (that is, if more than 70% of items referred to motor results); (3) principally focused on measuring behavior, cognition, or a child’s relationship with family members, peers, etc.; and (4) high-technology tools or devices or tools in the research stage or still under development without the support of scientific studies on the psychometric properties of the tests (see Tables 2 and 3).

**Table 3:** **Summary table**.

Tools	Available	Experimental phase	Age of application	Proxy methodology	Test methodology	Modulation assess	Perception and discrimination assess				Praxis assess	Language available
									
							Vestibular	Proprioceptive	Visual	Tactile		
SIPT	✓		4–9		✓		✓	✓	✓	✓	✓	English
SP^2^	✓		0–14.11	✓		✓	✓	✓	✓	✓		English and Spanish
SPM	✓		2–5 SPM-P 5-12 SPM	✓		✓	✓	✓	✓	✓	✓	English, Danish, Finnish, and Swedish
DeGangi–Berk Test of Sensory Integration (58)	✓		3–5		✓		✓	✓	✓		✓	English
Touch Inventory for elementary School-aged Children (TIE) (61, 62)	✓		6–12	Self-report and parents–school questionnaire (TIP)		✓				✓		English and Hebrew
Sensorimotor clinical observations (63–66)	✓		From 5		✓		✓	✓	✓		✓	English and Spanish
Comprehensive Observations of Proprioception^a^ (66)	✓		From 2					✓				English and Spanish
Miller Assessment Preschoolers (68)	✓		2.9–5.8		✓	✓	✓	✓	✓		✓	English, Japanese and Hebrew
Sensory Experiences Questionnaire Version 3.0 (SEQ-3.0)^a^ (7, 69–72)	✓		From 2 to 12 years SEQ from 6-72 months	✓		✓						English
The Sensory Processing Scales version 2.0 (28)		✓	From 4–19			✓						English
Test of Ideational Praxis (73)		✓	From 5–8 and preschooler form								✓	English
Pediatric Clinical Test of Sensory Interaction for Balance (79)	✓		Over 6		✓		✓	✓	✓			English
Classroom Sensory Environment Assessment^a^ (75)	✓		From 6		✓	✓						English
Preschool Imitation and Praxis Scale^a^	✓		1.5–4.9			✓					✓	English
Sensory Processing Assessment (SPA) (43, 80)	✓		3–5		✓	✓			✓	✓		English
Motor Planning Maze Assessment^a^ (73)	✓		3–5		✓						✓	English

*^a^These tools have been created, but researchers are still conducting further investigations with an enlarged sample to improve validity and reliability*.

### Data Collection

Once the assessment tools were identified, we administered the PRISMA checklist for systematic review (4) (see Table 1) and the Osteba Critical Appraisal Cards (FLC; http://www.lecturacritica.com/es/) (76). We also evaluated the clinical use, reliability, validity, assessment type (referring to a criterion or not referring to standards), target age, and study sample characteristics.

## Results

### Assessment Tools

Among the articles reviewed, 24 available tools for evaluating sensory processing in children between 3 and 11 years, independent of each clinical condition, were identified. Specifically, among these tools, 11 were experimental or were supported by few published studies on psychometric characteristics (see Table 2). The instruments that evaluate modulation do so mainly through proxy methodology, that is, through questionnaires provided to caregivers, parents, or teachers. Most of the instruments available to assess discrimination, SI, and praxis are tests instead of questionnaires.

According to our review, the tools most commonly used to determine sensory processing include the Sensory and Integration Praxis Tests (SIPT); the Sensory Profile (SP, or the more recent SP^2^ version) (44, 45), which features different formats for different age groups [short sensory profile, sensory profile for children, teacher sensory profile questionnaires (6, 54), and the Sensory Processing Measure (SPM) (52)] in combination with sensorimotor observation (67).

### Description of Included Tools

The SIPT is the “*gold standard*” measure for assessing sensory discrimination and sensorimotor disorders (6, 7, 35, 81–84). The test is a battery of 17 subtests designed to assess four factors: (1) tactile processing and discrimination; (2) vestibular and proprioceptive processing; (3) praxis and bilateral integration and sequencing; and (4) perception of shape and space and visuomotor coordination. The SIPT has been criticized for not providing information on the existence of SMD (28). It is also worth noting that this test is confined to use with children aged between 4 years and 8 years 11 months.

The Miller Assessment for Preschoolers (MAP) (68) was designed to provide a profile of sensory discrimination abilities, postural foundations, and praxis. In addition, it screens for visual, perceptual, and language delays that could be affecting participation in the classroom. The MAP offers an alternative to the SIPT as it involves less complex instructions and shorter subtest tasks and does not require certification to be administered (85).

Two tools were found for the assessment of sensory modulation in different populations: the Sensory Profile and the Sensory Processing Measure. The Sensory Profile (SP and SP^2^) is based on Dunn’s sensory processing model (54). There are two key factors in this model: the neurological threshold, which refers to the amount of stimuli required for a neuron or neuron system to respond, and the type of self-regulatory response exhibited by the child (45, 54). At the extreme ends of the neurological threshold are habituation (related to high thresholds) and sensitization (related to low thresholds). Kandel (86) identified several cellular mechanisms of learning that have been applied in the study of sensory processing: habituation, sensitization. Habituation is the simplest form of implicit learning, through which the properties of a new stimulus become familiar. Attention occurs when a new stimulus occurs for the first time. When the stimulus becomes familiar, or is neither beneficial nor harmful, there is no need to attend to the stimulus and so habituation occurs. Sensitization is the process that enhances the awareness of important stimuli. The central nervous system recognizes the stimuli as important or potentially harmful and generates a heightened response. Sensitization can sometimes be associated with anatomical changes, such as an increase in the number of neuron connections available for a task. Sensitization is a more complex mechanism than habituation (86).

The ability to modulate responses of the nervous system or maintain the balance between high and low thresholds allows a child to notice enough stimuli to be aware and attentive, thus avoiding an excess of information that could overload or distract. On the other hand, self-regulation is the ability of individuals to change their behavior under the demands of specific situations. Both actions are considered to be part of the learning process of the central nervous system.

The neurological threshold and self-regulation continua can help explain children’s performance based on four sensory processing patterns: (1) registration/bystander; (2) sensitivity/sensor; (3) avoiding/avoider; and (4) seeking/seeker. Registration represents high neurological threshold with passive self-regulation. Seeking represents high neurological thresholds but involves an active self-regulation strategy and the generation of new ideas. Sensitivity represents low neurological thresholds and a passive self-regulation strategy. Finally, avoiding represents low neurological thresholds with an active self-regulation strategy (1) (p.12).

The Sensory Profile (SP and SP^2^) comprises questionnaires for parents and teachers of children aged between birth and 14 years, although the authors later developed measures for adolescents and adults. There is also a teacher version (*School Companion*) that assesses four school factors: (1) the student’s need for external support to participate in learning activities, assessed through seeking and registration items; (2) awareness and attention within the learning environment, assessed through seeking and sensitivity items; (3) the student’s range of tolerance within the learning environment, assessed through avoiding and sensitivity items; and (4) the student’s availability for learning in the classroom, assessed through avoiding and registration items (54). Furthermore, the SP^2^ provides guidelines for intervention that focus on environmental strategies.

The Sensory Processing Measure (SPM home form; and SPM school environments form) (52, 87) is a questionnaire evolving from two previous measures: the *Evaluation of Sensory Processing* (ESP) and the *School Assessment of Sensory Integration* (SASI). There is also a version for preschoolers (SPM-P). The form for children aged from 3 to 10 years comprises 62 items distributed across different domains: social participation, vision, hearing, touch, body awareness, balance and motion, and motor planning (52, 87). SPM is a tool for evaluating elements related to sensory processing, praxis, and social participation in different school environments. The aim of this tool is to provide teachers with information regarding sensory facilitators and barriers to help students perform successfully. Pilot studies suggest that SPM-School is a reliable and valid tool. However, results have shown that the sensory processing items exhibit lower internal consistency than the social participation items. Validity has been observed to be higher when discriminating between children with and without sensory processing issues (88). The tool has been cross-culturally translated to Danish, Finnish, Norwegian, Swedish, and Chinese. Lai et al. observed the patterns of behavioral response toward sensory stimuli in the Hong Kong population with the Sensory Processing Measure Chinese version. These differences suggest the importance of the child’s environment. The findings showed that the Sensory Processing Measure-Hong Kong Chinese version was a valid and reliable tool in the screening for sensory processing of children aged 5–12 among Chinese populations (53).

The Touch Inventory for Elementary School-Aged Children (TIE) (61) is a children’s self-report measure of tactile defensiveness; authors recommend that the TIE be used in conjunction with the modified parental version of the TIE to supplement and identify more clearly the family contexts in which children live and to support family-based/client-centered therapy and outcomes. More extensive research studies addressing construct validity, clinical utility, and responsiveness must be completed (89). Furthermore, the author developed a preschool version that could be applicable to children who are developmentally delayed and non-verbal children (61).

The Sensory Experience Questionnaire 3.0 (SEQ-3.0) (69), is useful in obtaining sensory characteristics and discriminating sensory patterns of hypo- and hyper-responsiveness among children with autism and mental or developmental retardation between 2 and 12 years old.

Another questionnaire, the Sensory Sensitivity Questionnaire-Revised, is oriented toward determining whether children with autism show sensory hyper- and hypo-sensitivities in six areas: auditory, visual, tactile, gustatory, vestibular, and olfactory (90).

In addition, the evaluation of sensorimotor disorders can be conducted through clinical observations, which are principally aimed at detecting vestibular, proprioceptive, and/or proprioceptive/vestibular difficulties (65, 67). The Clinical Observations of Motor and Postural Skills could provide additional insight into the maturity of the child’s nervous system, as well as rich qualitative observations of sensory discrimination, muscle tone, strength, sequencing, and planning (85). The tool allows for observational assessment and helps interpret behaviors that may be related to proprioception during skilled motor learning tasks and everyday tasks, such as sitting posture, balance responses, and use of body during play (67). Structured and non-structured clinical observations are a useful tool for evaluating children who, because of their age or diagnosis, cannot be assessed using other tools. Structured clinical observations measure the following functions:

(a) Vestibular processing, which includes *vestibulo-spinal* function (balance reactions, extensor muscle tone, and neck and upper trunk stability); *vestibulo-ocular* function (capacity to conduct visual tracking and maintain a stable field of vision); *vestibulo-perceptual* function (spatial orientation, spatial memory, and the ability to move in space); *excitability* of the vestibular system; *anticipatory mechanisms;* and *bilateral coordination*.
(b) Proprioceptive processing, which includes *spinal function* (muscle tone, stretching reflexes, and dynamic stability); *subcortical functions* (posture control and fluidity of movement); *cortical functions* (awareness of the position of joints and motor planning). Proprioception is also closely related to excitement control and must be assessed.
(c) Vestibular-proprioceptive, which includes *posture control* and *anticipatory mechanisms*.

The Comprehensive Observations of Proprioception (COP) is a new assessment tool that organizes observations to provide a structured method for assessing the relationship between proprioceptive information and motor performance (postural control, motor planning, and proximal stability), as well as level arousal modulation. The aim is to identify proprioceptive processing disorders in children with developmental disabilities, and the tool is used in combination with clinical sensory–motor observations or when the child is observed during free play. Validity was established between the COP results and the results of items from the SPM (body awareness), the kinesthesia test, and the standing and walking balance test from the SIPT. Results of factor analysis revealed four groups of proprioceptive dysfunctions: (a) muscle tone and proximal joint stability; (b) behavioral manifestations of proprioceptive seeking; (c) postural control; and (d) motor planning (67).

Table 2 shows the different tools that can be included in an assessment to evaluate sensory processing dysfunction. Additionally, Table 3 summarizes the results obtained.

### Excluded Tools

According to our previously established criteria, we excluded the use of other instruments focused mainly on development, visual, or motor skills. We also excluded tools used in other approaches and environments, such as developmental psychology, neurophysiology, and neuropsychology. In this respect, we excluded 10 tests: Bruininks-Oseretsky Test of Motor Proficiency (91); Bayley Scales of Infant Development-III (92, 93); Movement Assessment Battery for Children 2 (94); Batelle Developmental Inventory (95); Peabody Developmental Motor Scales (96); Test of Visual–Motor Skills-3m (97); Developmental Test of Visual Perception (DTVP) (98); Developmental Test of Visual–Motor Integration sixth ed (99).; Test of Visual Perceptual Skills (TVPS-3) (100) and Motor-Free Visual Perception Test (101); and the Developmental Coordination Disorder Questionnaire (102) (see Annex S1 in Supplementary Material) (103–107).

We also excluded other tactile assessment tools designed for specific populations (36) and electroencephalography (EEG), which is used to diagnose SPDs (108). In addition, we excluded other instruments that are commercially available but for which scientific studies have not consulted databases on the psychometric properties of the tests employed or, in some cases, for which no standardized methods are provided for assessment. This group includes (1) the Preschool Sensory Scan for Educators (109), which is a checklist designed for teachers to identify children who they feel may be at risk for SPD under three categories: sensory modulation, sensory discrimination, and sensory-based motor skills. Each of these categories focuses on how the senses (tactile, vestibular, proprioceptive, visual, auditory, and olfactory) are affected. The instrument is available only for children between 2½ and 5 years. A list of primary and secondary therapies is also included. The tool is available only for the US population. The group also includes (2) the Quick Neurological Screening Test-third Edition (110), which is available only in English for persons between 5 and 80 years old. The principal aim is to assess neurological soft signs. Additionally, there is (3) Sensorimotor Performance Analysis (111). This tool consists of four gross motor tasks and three fine motor tasks that are broken down by performance components. Although developed specifically for cognitively handicapped, school-aged clients, SPA has been found useful for clients in other age groups and clients with a variety of sensorimotor problems, including dysfunction in postural control and movement patterns. The instrument is available only in English for individuals aged 5 years to adult. Furthermore, there is (4) the Sensory Integration Inventory Revised for Individuals with Developmental Disabilities (SII-R) (112). The inventory was designed to screen for clients with developmental delays and disabilities who might benefit from a SI treatment approach and is a non-standardized checklist. Finally, there is (5) Sensory Processing Assessment (SPA) (43), a play-based behavioral observation assessment that allows for the detection of hypersensitivity to specified sensory stimuli. The assessment is specially designed to test children with autism and has been used to assess sensory interests, repetitions, and seeking behaviors (SIRS) (80). The checklist is not standardized and is related to research rather than to clinical practice.

## Discussion, Limitations, and Conclusion

To the best of our knowledge, this is the first systematic review of tools that are useful in assessing sensory processing in children between 3 and 11 years. Additionally, we have included the languages in which each instrument is available. This study may help establish future goals to meet the needs that exist in the evaluation of sensory processing.

According to Roley et al. (81), there are certain groups that require comprehensive evaluation of sensory processing interest, such as children with ASD (81). We also believe that somatosensory evaluation and praxis would be very useful in children with ADHD according to recent neuroimaging studies and other studies in this field (113–115).

The results of this systematic review reveal that there are a total of 21 tools available for the evaluation of the different stages of sensory processing in children aged between 3 and 11 years. Among these, 15 tests are available and are supported by psychometric studies, primarily for the US population. Nine of the tests can be applied to children in preschool to grade 12. Only three of them are designed solely for preschool children. Other tools feature newly developed tests or questionnaires and research processes. Among all tests, eight provide insight into the process of modulation, nine provide information about the process of discrimination, and eight allow for the assessment of praxis.

Most tests are only available in English and are designed for the US population. However, the two main tools for assessing modulation are available in different languages. Specifically, they are six versions of SPM (English, Danish, Finish, Swedish, Norwegian, and Chinese) and six different versions of SP (English, Spanish, Arabic, Turkish, Indian, and Chinese). Unfortunately, the SITP is only available in English and is designed for the North American population.

The SIPT is the main comprehensive test with objective tasks for evaluating sensory processing. Asher et al. reported high reliability for SIPT scores in determining the presence of SI disorder. Nevertheless, additional information is needed for a more reliable interpretation of SIPT scores, such as clinical observations and case history, to help clinicians make the more subtle distinctions needed to determine the relevance of the different sensory features for each case (35). However, the SIPT does have some disadvantages. The test has only been validated in a North American population, which limits its application to other populations. Furthermore, it has never been revised since it was created in 1989. Examiners need to be accredited to administer the test, and both the training and the test itself are costly. Another limitation is the length of time needed to administer and correct the test, which means it is not frequently used in daily clinical practice. Indeed, Szklut (85) recommends the use of the MAP rather than the SIPT in children under the age of 6 because of the ease of access to the test, its lower cost, and the fact that the items are aimed at preschoolers and the test is easier to correct (85). The results of our systematic review can be helpful and promote interest in new sensory processing evaluation tests. Given the potential usefulness and thoroughness of the test, it would be useful to have an updated version of the SIPT, given the best available evidence to assess proprioceptive, vestibular, and tactile sensory discrimination and praxis, which may apply to a broader age range, i.e., to children between 3 and 11 years.

The SP (and SP^2^, the updated version) and the SPM are two complementary questionnaires for assessing sensory modulation using information from parents and teachers. These tools help to detect the presence of modulation differences, although they do not permit identification of discrimination dysfunction, for which clinical tests or observations are required. Both tools enable the detection of sensory processing problems in children within their school environment (88). However, Lai et al. reported that although the SPM-School was a highly reliable and valid tool when used with children aged 5 to 12, they recommended using complementary tools for assessing other settings because the correlation between the Home Form and the Classroom Form was low (53). One advantage of these tools is that they allow data to be collected rapidly, even electronically. Furthermore, there exists a shorter version of the SP, the SSP, which has demonstrated discriminate validity of over 95% in identifying children with and without sensory modulation differences (57), which makes it especially useful as a screening method.

A significant difference between the two questionnaires is that SPM provides information on social participation and praxis, whereas the SP^2^ analyzes children’s neurological threshold and responses associated with emotional and behavioral self-regulation throughout their daily life (7).

It is worth noting that specific, exhaustive protocols are being developed for the use of sensorimotor clinical observations as wider-ranging tests in the assessment of the proprioceptive and vestibular systems. For example, the COP provides a reliable measure for identifying the origin of proprioceptive difficulties that affect children’s functional performance. Inter-rater reliability is high (0.91), and the tool is easy to administer (67).

The results of our study differ from those obtained in the systematic review conducted by Eeles et al. to identify the tools available for measuring sensory processing in children aged 0 to 3 years (4). These authors found that the Sensory Profile (SP) enables early detection of possible modulation or regulation disorders in early childhood. Therefore, we can conclude that, in contrast to the case of the first 3 years of life, in addition to clinical observations and questionnaires, there are also specific tests and tasks for assessing sensory processing designed for the 3–11 age group.

There are still areas in which assessment tools need to be developed, such as the evaluation of overall tactile processing. In this respect, Auld et al. review different clinically useful tools for the assessment of tactile SI, especially for evaluating registration and perception (36). In addition, it could be interesting to develop new assessments of pain for children.

Sensory integration difficulties affect the daily life and functionality of children with dysfunction. Early detection of these particular aspects of SI and praxis will help researchers design specific treatment programs (7). Anomalies in the modulation of one or more sensory channels is one of the first signs of alarm detected by parents, even at very early ages, as in the case of autism spectrum disorders. The close link between the different anomalies of SI and neurodevelopmental disorders in early childhood make assessing sensory processing especially relevant (7, 116). New assessment tools or an updated version of SPIT are required to ensure correct diagnosis of the sensory and motor factors that can affect functionality and participation in daily life activities during childhood, especially for children aged 3 and 4 and children over the age of 9. Key areas to be developed include measures of sensory modulation and wider-ranging tools covering measures of proprioception and vestibular function, standardized assessments of posture and balance and specific measures of praxis (especially ideation and motor planning).

Our review reveals an increase over recent years in the number of tools for measuring sensory processing, both those that are fully validated and those in the research stage. The number has risen from one study published per year between 2006 and 2009, to seven in 2014, demonstrating a trend toward prioritizing the assessment of sensory processing because of its relationship with difficulties in development, learning, and behavior in childhood.

This systematic review shows some limitations that should be considered when interpreting the findings. First, sample exhaustivity: the review article only draws upon relevant studies published in English and Spanish language according to specified search criteria. Second, cultural biases: most of assessment tools referred in our review have been designed in North American context and tested out with North American samples.

Despite such limitations, we consider this systematic review shows relevant information that could help in making decisions about what assessment tools are available and what are more accurate for each age and different patterns of sensory processing. It is the first systematic review focused on Assessment of Sensory Processing Characteristics in Children between 3 and 11 years old. We hope this review will boost pediatricians, neurologists, and occupational therapists to take into account this mode of assessment in their daily clinical practice, in particular when assessing ASD and others neurodevelopmental disorders that could help to an early identification of SPDs: modulation, sensory-based motor, and sensory discrimination disorders (11–19).

Not all cultures have specific instruments for the assessment of all dimensions of sensory processing. In these cases, the use of standardized instruments for the target population may be useful for the assessment of specific dimensions required for sensory processing, such as fine and gross motor skills, motor planning, praxis, sequencing, fluidity, and control of movement, particularly children where a sensory-based motor disorders are suspected. In this sense, it could be useful Bruininks-Oseretsky Test of Motor Proficiency, Bayley Scales of Infant and Toddler Development Motor Scale (BSID) among others, to assess motor control, or TVPS-3 or DTVP to assess visual perception, or NEPSY-2 with the aim to know praxis. However, given that the most prevalent SPD are SMDs (43%) (88), in addition to these instruments, SP^2^ or SPM might be used to assess SMDs, especially in children born preterm and in low birthweight, because they are at risk to suffer developmental disorders (53, 61). In this way, the early detection of SPD is considered basic with the aim to improve the development and the adaptive behavior in childhood.

Transculturally adapted studies are thus a priority to permit the identification of SPDs in other populations and thereby facilitate access to treatment of infant neurodevelopmental disorders. An effective future approach to the assessment of sensory processing may well lie in the fusion of standardized tests with neurophysiological tests, which could permit the use of computerized tasks and brain-imaging techniques such as MEG and RMN (7, 24, 117, 118).

Several important themes regarding assessment and future research in the area of SI and sensory processing emerged from this review. First, it is necessary to develop objective tests to evaluate the modulation in addition to proxy methodology. Second, it is important develop new tools to assess sensory discrimination in children between 0 and 4 years, as well as for children over 9 years old through adolescence. Third, of all the evidence analyzed, none can tell us whether their recording and sensory quality assessment have been performed, which is why it would be desirable to incorporate these elements into the evaluation process, especially in research-based, objective assessment tools *via* EEG, TMS, and neuroimaging techniques that allow researchers to check how it has produced the sensory register. Fourth, there is a lack of tools that help the clinician determine tactile sensory characteristics, such as the processing of pain, taste, and auditory stimuli.

Finally, although there is evidence of the effectiveness of SI therapy, in the future, randomized controlled trials, systematic reviews, and meta-analyses for different population groups (ASD, ADHD, and other neurodevelopmental disorders or perinatal conditions) should be performed to continue strengthening the effectiveness of occupational therapy using SI.

## Author Contributions

All authors conducted the search of literature, reviewed the articles, helped with data synthesis and interpretation, and played a major role in writing the manuscript.

## Conflict of Interest Statement

The authors declare that the research was conducted in the absence of any commercial or financial relationships that could be construed as a potential conflict of interest.

## References

[1] Dunn W Sensory Profile 2: User’s Manual. USA: Pearson, Inc (2014).

[2] Dunn W Supporting children to participate successfully in everyday life by using sensory processing knowledge. Infant Young Child (2007) 20(2):84–101. 10.1097/01.IYC.0000264477.05076.5d

[3] Yack E Sutton S Aquilla P Building Bridges through Sensory Integration. Las Vegas, NV: Sensory Resources (2002).

[4] Eeles A Spittle AJ Anderson PJ Brown N Lee K Boyd R Assessment of sensory processing in infant: a systematic review. Dev Med Child Neurol (2013) 55:314–26. 10.1111/j.1469-8749.2012.04434.x 23157488

[5] Crozier SC Goodson JZ Mackay ML Synnes AR Grunau RE Miller SP Sensory processing patterns in children born very preterm. Am J Occup Ther (2015) 70(1):7001220050p1–7. 10.5014/ajot.2016.018747 26709425

[6] Ayres J Sensory Integration and Praxis Tests (SPIT). Los Angeles: Western Psychological Services (1989).

[7] Schaaf RC Lane AE Toward a best-practice protocol for assessment of sensory features in ASD. J Autism Dev Disord (2015) 45(5):1380–95. 10.1007/s10803-014-2299-z 25374136

[8] Brown T Morrison IC Stagnitti K The convergent validity of two sensory processing scales used with school-age children: comparing the Sensory Profile and the Sensory Processing Measure. New Zeal J Occup Ther (2010) 57(2):56–65.

[9] Baranek G Little LM Parham D Ausderau KK Sabatos-De Vito MG Sensory features in autism spectrum disorders. 4th ed In: Volkmar FR Rogers SJ Paul R Pelphrey KA, editors. Handbook of Autism and Pervasive Developmental Disorders. New Jersey: John Wiley & Sons, Inc (2014). p. 378–408.

[10] Ayres AJ Tickle LS Hyper-responsivity to touch and vestibular stimuli as a predictor of positive response to sensory integration procedures by autistic children. Am J Occup Ther (1980) 34(6):375–81. 6971048 10.5014/ajot.34.6.375

[11] Ayres AJ Effect of sensory integrative therapy on the coordination of children with choreoathetoid movements. Am J Occup Ther (1977) 31(5):291–3. 860743

[12] Ayres AJ Cluster analyses of measures of sensory integration. Am J Occup Ther (1977) 31(6):362–6. 879252

[13] Bowyer P Cahill SM Pediatric Occupational Therapy Handbook. A Guide to Diagnoses and Evidence-Based Practice. St. Louis: Mosby Elsevier (2009).

[14] Parham LD Mailloux Z Sensory integration. 4th ed In: Case-Smith J, editor. Occupational Therapy for Children. St. Louis: Mosby (2001). p. 329–81.

[15] Ahn RR Miller LJ Milberger S McIntosh DN Prevalence of parents’ perceptions of sensory processing disorders among kindergarten children. Am J Occup Ther (2004) 58(3):287–93. 10.5014/ajot.58.3.287 15202626

[16] Dunn W Sensory Profile Supplement User’s Manual. San Antonio, TX: Harcourt Assessment (2006).

[17] Baranek GT Chin YH Hess LM Yankee JG Hatton DD Hooper SR Sensory processing correlates of occupational performance in children with fragile X syndrome: preliminary findings. Am J Occup Ther (2002) 56(5):538–46. 10.5014/ajot.56.5.538 12269508

[18] Kern JK Garver CR Carmody T Andrews AA Trivedi MH Mehta JA Examining sensory quadrants in autism. Res Autism Spectr Disord (2007) 1(2):185–93. 10.1016/j.rasd.2006.09.002

[19] Reebye P Stalker A Understanding Regulation Disorders of Sensory Processing in Children: Management Strategies for Parents and Professionals. London: Jessica Kingsley Publishers (2008).

[20] Rogers SJ Hepburn S Wehner E Parent reports of sensory symptoms in toddlers with autism and those with other developmental disorders. J Autism Dev Disord (2003) 33(6):631–42. 10.1023/B:JADD.0000006000.38991.a7 14714932

[21] ICDL. Diagnostic Manual for Infancy and Early Childhood. Bethesda: ICDL (2005).

[22] Eapen V Črnčec R DSM 5 and child psychiatric disorders: what is new? What has changed? Asian J Psychiatr (2014) 11:114–8. 10.1016/j.ajp.2014.04.008 25453713

[23] Miller LJ Anzalone ME Lane SJ Cermak SA Osten ET Concept evolution in sensory integration: a proposed nosology for diagnosis. Am J Occup Ther (2007) 61(2):135–40. 10.5014/ajot.61.2.135 17436834

[24] Miller LJ Nielsen DM Schoen SA Attention deficit hyperactivity disorder and sensory modulation disorder: a comparison of behavior and physiology. Res Dev Disabil (2012) 33(3):804–18. 10.1016/j.ridd.2011.12.005 22236629

[25] Dunn W Myles BS Orr S Sensory processing issues associated with Asperger syndrome: a preliminary investigation. Am J Occup Ther (2002) 56(1):97–102. 10.5014/ajot.56.1.97 11833406

[26] Miller LJ Schoen SA James K Schaaf RC Lessons learned: a pilot study on occupational therapy effectiveness for children with sensory modulation disorder. Am J Occup Ther (2007) 61(2):161–9. 10.5014/ajot.61.2.161 17436838

[27] Blanche EI Parham D Chang M Mallinson T Development of an adult sensory processing scale (ASPS). Am J Occup Ther (2014) 68(5):531–8. 10.5014/ajot.2014.012484 25184465

[28] Schoen SA Miller LJ Sullivan JC Measurement in sensory modulation: the sensory processing scale assessment. Am J Occup Ther (2014) 68(5):522–30. 10.5014/ajot.2014.012377 25184464 PMC4153553

[29] Goldsmith HH Van Hulle CA Arneson CL Schreiber JE Gernsbacher MA A population-based twin study of parentally reported tactile and auditory defensiveness in young children. J Abnorm Child Psychol (2006) 34(3):393–407. 10.1007/s10802-006-90240 16649001 PMC4301432

[30] Dunn W The sensations of everyday life: empirical, theoretical, and pragmatic considerations. Am J Occup Ther (2001) 55(6):608–20. 10.5014/ajot.55.6.608 12959225

[31] Dunn W Brown C Factor analysis on the Sensory Profile from a national sample of children without disabilities. Am J Occup Ther (1997) 51(7):490–5; discussion 6–9. 9242854 10.5014/ajot.51.7.490

[32] Mailloux Z Mulligan S Roley SS Blanche E Cermak S Coleman GG Verification and clarification of patterns of sensory integrative dysfunction. Am J Occup Ther (2011) 65(2):143–51. 10.5014/ajot.2011.000752 21476361

[33] Lane SJ Bundy AC Kids Can Be Kids a childhoods Occupations Approach. Philadelphia: F.A Davis Company (2012). p. 437–59.

[34] Miller LJ Coll JR Schoen SA A randomized controlled pilot study of the effectiveness of occupational therapy for children with sensory modulation disorder. Am J Occup Ther (2007) 61(2):228–38. 10.5014/ajot.61.2.228 17436845

[35] Asher AV Parham LD Knox S Interrater reliability of Sensory Integration and Praxis Tests (SIPT) score interpretation. Am J Occup Ther (2008) 62(3):308–19. 10.5014/ajot.62.3.308 18557007

[36] Auld ML Boyd RN Moseley GL Johnston LM Tactile assessment in children with cerebral palsy: a clinimetric review. Phys Occup Ther Pediatr (2011) 31(4):413–39. 10.3109/01942638.2011.572150 21599569

[37] Parham LD Roley SS May-Benson TA Koomar J Brett-Green B Burke JP Development of a fidelity measure for research on the effectiveness of the Ayres Sensory Integration intervention. Am J Occup Ther (2011) 65(2):133–42. 10.5014/ajot.2011.000745 21476360

[38] Schaaf RC Burke JP Cohn E May-Benson TA Schoen SA Roley SS State of measurement in occupational therapy using sensory integration. Am J Occup Ther (2014) 68(5):e149–53. 10.5014/ajot.2014.012526 25184475

[39] Mailloux Z May-Benson TA Summers CA Miller LJ Brett-Green B Burke JP Goal attainment scaling as a measure of meaningful outcomes for children with sensory integration disorders. Am J Occup Ther (2007) 61(2):254–9. 10.5014/ajot.61.2.254 17436848

[40] Reuben DB Magasi S McCreath HE Bohannon RW Wang YC Bubela DJ Motor assessment using the NIH Toolbox. Neurology (2013) 80(11 Suppl 3):S65–75. 10.1212/WNL.0b013e3182872e01 23479547 PMC3662336

[41] Koenig KP Rudney SG Performance challenges for children and adolescents with difficulty processing and integrating sensory information: a systematic review. Am J Occup Ther (2010) 64(3):430–42. 10.5014/ajot.2010.09073 20608274

[42] Baranek GT Efficacy of sensory and motor interventions for children with autism. J Autism Dev Disord (2002) 32(5):397–422. 10.1023/A:1020541906063 12463517

[43] Baranek GT Boyd BA Poe MD David FJ Watson LR Hyperresponsive sensory patterns in young children with autism, developmental delay, and typical development. Am J Ment Retard (2007) 112(4):233–45. 10.1352/0895-8017(2007)112%5B233:HSPIYC%5D2.0.CO;2 17559291

[44] Dunn W Westman K The sensory profile: the performance of a national sample of children without disabilities. Am J Occup Ther (1997) 51(1):25–34. 8978860 10.5014/ajot.51.1.25

[45] Dunn W Performance of typical children on the sensory profile: an item analysis. Am J Occup Ther (1994) 48(11):967–74. 7530904 10.5014/ajot.48.11.967

[46] Wei BY Wei YY Huang F [Influential factors for the sensory integration training effects in children with autism]. Zhongguo Dang Dai Er Ke Za Zhi (2009) 11(2):124–7. 19222950

[47] McIntosh DN Miller LJ Shyu V Hagerman RJ Sensory-modulation disruption, electrodermal responses, and functional behaviors. Dev Med Child Neurol (1999) 41(9):608–15. 10503919 10.1017/s0012162299001267

[48] Bundy A Lane SJ Fisher A Sensory Integration: Theory and Practice. Philadelphia: F.A. Davis (2002).

[49] Provost B Oetter P The sensory rating scale for infants and young children: development and reliability. Phys Occup Ther Pediatr (1993) 13(4):15–35.

[50] Dunn W Infant/Toddler Sensory Profile. User’s Manual. San Antonio, TX: The Psychological Corporation (2002).

[51] Degangi G Greenspan SI Test of Sensory Functions in Infants (TSFI) Manual. Los Angeles: CA: Western Psychological Services (1989).

[52] Parham LD Ecker C Miller H Henry DA Glennon TJ Sensory Processing Measure. Los Angeles: WPS (2007).

[53] Lai CY Chung JC Chan CC Li-Tsang CW Sensory processing measure-HK Chinese version: psychometric properties and pattern of response across environments. Res Dev Disabil (2011) 32(6):2636–43. 10.1016/j.ridd.2011.06.010 21752596

[54] Dunn W The Sensory Profile: User’s Manual. San Antonio: The Psychological Corporation (1999).

[55] Abu-Dahab SM Malkawi SH Nadar MS Al Momani F Holm MB The validity and reliability of the Arabic infant/toddler sensory profile. Phys Occup Ther Pediatr (2014) 34(3):300–12. 10.3109/01942638.2013.823474 23931241

[56] Benjamin TE Crasta JE Suresh AP Alwinesh MJ Kanniappan G Padankatti SM Sensory profile caregiver questionnaire: a measure for sensory impairment among children with developmental disabilities in India. Indian J Pediatr (2014) 81(Suppl 2):S183–6. 10.1007/s12098-014-1603-4 25338495

[57] Tomchek SD Dunn W Sensory processing in children with and without autism: a comparative study using the short sensory profile. Am J Occup Ther (2007) 61(2):190–200. 10.5014/ajot.61.2.190 17436841

[58] DeGangi GA Berk R DeGangi-Berk Test of Sensory Integration (TSI) Manual. Los Angeles, CA: Western Psychological Services (1983).

[59] DeGangi GA Berk RA Larsen LA The measurement of vestibular-based functions in pre-school children. Am J Occup Ther (1980) 34(7):452–9. 6967251 10.5014/ajot.34.7.452

[60] Berk RA DeGangi GA Technical considerations in the evaluation of pediatric motor scales. Am J Occup Ther (1979) 33(4):240–4. 155983

[61] Royeen CB The development of a touch scale for elementary school aged children. Am J Occup Ther (1986) 40:414–9. 3717276 10.5014/ajot.40.6.414

[62] Royeen CB Fortune JC Touch inventory for elementary-school-aged children. Am J Occup Ther (1990) 44(2):155–9. 1689963 10.5014/ajot.44.2.155

[63] Ayres AJ Interpreting the Southern California Sensory Integration Test. Los Angeles, CA: Western Psychological Services. WPS (1984).

[64] Blanche E Observations Based on Sensory Integration. Torrance, CA: Pediatric Therapy Network (2002).

[65] Blanche EI Observations Based on Sensory Integration Theory. Torrance, CA: Pediatric Therapy Network (2010).

[66] Blanche E Reinoso G Blanche-Kiefer D Observaciones clínicas sensoriomotoras. Evaluaciones y aplicación clínica en niños con dificultades en el desarrollo y procesamiento sensorial. Los Angeles, CA: Sensory Metrics, Inc (2014).

[67] Blanche EI Bodison S Chang MC Reinoso G Development of the comprehensive observations of proprioception (COP): validity, reliability, and factor analysis. Am J Occup Ther (2012) 66(6):691–8. 10.5014/ajot.2012.003608 23106989 PMC3756186

[68] Miller LJ Miller Assessment for Preschoolers Manual (Revised Edition). San Antonio, TX: Psychological Corporation (1988).

[69] Baranek GT David FJ Poe MD Stone WL Watson LR Sensory experiences questionnaire: discriminating sensory features in young children with autism, developmental delays, and typical development. J Child Psychol Psychiatry (2006) 47(6):591–601. 10.1111/j.1469-7610.2005.01546.x 16712636

[70] Little LM Freuler AC Houser MB Guckian L Carbine K David FJ Psychometric validation of the sensory experiences questionnaire. Am J Occup Ther (2011) 65(2):207–10. 10.5014/ajot.2011.000844 21476368 PMC3163482

[71] Little LM Sideris J Ausderau K Baranek GT Activity participation among children with autism spectrum disorder. Am J Occup Ther (2014) 68(2):177–85. 10.5014/ajot.2014.009894 24581404 PMC4012568

[72] Ausderau K Sideris J Furlong M Little LM Bulluck J Baranek GT National survey of sensory features in children with ASD: Factor structure of the sensory experience questionnaire (3.0). Journal of autism and developmental disorders (2014) 44(4):915–25. 10.1007/s10803-013-1945-1 24097141 PMC3949144

[73] Ivey CK Lane SJ May-Benson TA Interrater reliability and developmental norms in preschoolers for the motor planning maze assessment (MPMA). Am J Occup Ther (2014) 68(5):539–45. 10.5014/ajot.2014.012468 25184466

[74] Crowe TK Deitz JC Richardson PK Atwater SW Interrater reliability of the pediatric clinical test of sensory interaction for balance. Phys Occup Ther Pediatr (1991) 10(4):1–27.

[75] Kuhaneck HM Kelleher J Development of the Classroom Sensory Environment Assessment (CSEA). Am J Occup Ther (2015) 69(6):6906180040p1–9. 10.5014/ajot.2015.019430 26565097

[76] Servicio de Evaluación de Tecnologías Sanitarias (Osteba). FLC Critica Manual de Uso: Versión 1.1.0. País Vasco: Servicio de Evaluación de Tecnologías Sanitarias (2008).

[77] Vanvuchelen M Roeyers H De Weerdt W Measuring procedural imitation aptitude in children: further validation of the preschool imitation and praxis scale (PIPS). Percept Mot Skills (2011) 113(3):773–92. 10.2466/10.11.22.PMS.113.6.773-792 22403923

[78] Vanvuchelen M Roeyers H De Weerdt W Objectivity and stability of the preschool imitation and praxis scale. Am J Occup Ther (2011) 65(5):569–77. 10.5014/ajot.2010.ajot00000414 22026325

[79] Richardson PK Atwater SW Crowe TK Deitz JC Performance of preschoolers on the pediatric clinical test of sensory interaction for balance. Am J Occup Ther (1992) 46(9):793–800. 1514565 10.5014/ajot.46.9.793

[80] Kirby AV Little LM Schultz B Baranek GT Observational characterization of sensory interests, repetitions, and seeking behaviors. Am J Occup Ther (2015) 69(3):6903220010p1–9. 10.5014/ajot.2015.015081 PMC5362027 25871592

[81] Roley SS Mailloux Z Parham LD Schaaf RC Lane CJ Cermak S Sensory integration and praxis patterns in children with autism. Am J Occup Ther (2014) 69(1):6901220010p1–8. 10.5014/ajot.2015.012476 25553746

[82] Mailloux Z An overview of sensory integration and praxis tests. Am J Occup Ther (1990) 44(7):589–94. 2386185 10.5014/ajot.44.7.589

[83] Cermak SA Murray EA The validity of the constructional subtests of the sensory integration and praxis tests. Am J Occup Ther (1991) 45(6):539–43. 2063943 10.5014/ajot.45.6.539

[84] Kimball JG Using the sensory integration and praxis tests to measure change: a pilot study. Am J Occup Ther (1990) 44(7):603–8. 2386187 10.5014/ajot.44.7.603

[85] Szklut S Using clinical reasoning to evaluate sensory processing dysfunction. Sens Integration Special Interest Section Q (2010) 33(4):1–4.

[86] Kandel ER Principles of Neural Science. 5th ed New York: McGraw-Hill Medical (2013). 1709 p.

[87] Henry D McClary M The sensory processing measure-preschool (SPM-P). Part two: test-retest and collective collaborative empowerment. Including a father’s perspective. J Occup Ther Schools Early Interv (2011) 4(1):53–70. 10.1080/19411243.2011.576891

[88] Miller-Kuhaneck H Henry DA Glennon TJ Mu K Development of the sensory processing measure-school: initial studies of reliability and validity. Am J Occup Ther (2007) 61(2):170–5. 10.5014/ajot.61.2.170 17436839

[89] Brown G Brown A A review and critique of the touch inventory for elementary school-aged children (TIE). Br J Occup Ther (2006) 69(5):234–43. 10.1177/030802260606900507

[90] Watling R Sensory Sensitivity Questionnaire-Revised (SSQ-R). Encyclopedia of Autism Spectrum Disorders. New York: Springer New York (2013). p. 2815–6.

[91] Bruininks R Bruininks B Bruininks-Oseretsky Test of Motor Proficiency. 2nd ed Minneapolis, MN: Pearson (2005).

[92] Bayley N Bayley Scales of Infant and Toddler Development. Third ed USA: Pearson (2005).

[93] Bayley N Escalas Bayley de desarrollo infantile – III. Adaptación española. Madrid: Pearson (2015).

[94] Henderson S Sudgen D Barnett A Movement Assessment Battery for Children 2-MABC. USA: Pearson (2007).

[95] Newborg J Battelle Developmental Inventory. 2nd ed Itasca, IL: Riverside Publishing (2004).

[96] Folio MR Fewell RR Peabody Developmental Scales. 2nd ed Austin: Pro-ed (2000).

[97] Martin N Test of Visual Motor Skills-3. Los Angeles, CA: WPS (2010).

[98] Hammil D Pearson N Voress J Developmental Test of Visual Perception (DTVP-3). USA: Pearson (2013).

[99] Beery KE Buktenica NA Beery NA Beery-Buktenica Developmental Test of Visual-Motor Integration. 6th ed Minneapolis, MN: Pearson (2010).

[100] Martin N Test of Visual Perceptual Skills-3 (TVPS-3). Novato, Canada: Academic Therapy Publications (2010).

[101] Colarusso R Hammill D Motor-Free Visual Perception Test. Novata, CA: Academic Therapy Publications (2003).

[102] Wilson BN Crawford SG Green D Roberts G Aylott A Kaplan B Psychometric properties of the revised developmental coordination disorder questionnaire. Phys Occup Ther Pediatr (2009) 29(2):182–202. 10.1080/01942630902784761 19401931

[103] McFall SA Deitz JC Crowe TK Test-retest reliability of the test of visual perceptual skills with children with learning disabilities. Am J Occup Ther (1993) 47(9):819–24. 8116773 10.5014/ajot.47.9.819

[104] Jirikowic TL Engel JM Deitz JC The test of sensory functions in infants: test-retest reliability for infants with developmental delays. Am J Occup Ther (1997) 51(9):733–8. 9311428 10.5014/ajot.51.9.733

[105] May-Benson TA Koomar JA Systematic review of the research evidence examining the effectiveness of interventions using a sensory integrative approach for children. Am J Occup Ther (2010) 64(3):403–14. 10.5014/ajot.2010.09071 20608272

[106] Lang R O’Reilly M Healy O Rispoli M Lyndon H Streusand W Sensory integration therapy for autism spectrum disorders: a systematic review. Res Autism Spectr Disord (2012) 6(3):1004–18. 10.1016/j.rasd.2012.01.006

[107] May-Benson TA Roley SS Mailloux Z Parham LD Koomar J Schaaf RC Interrater reliability and discriminative validity of the structural elements of the Ayres Sensory Integration Fidelity Measure. Am J Occup Ther (2014) 68(5):506–13. 10.5014/ajot.2014.010652 25184462 PMC4153552

[108] Lewine JD Davis JT Bigler ED Thoma R Hill D Funke M Objective documentation of traumatic brain injury subsequent to mild head trauma: multimodal brain imaging with MEG, SPECT, and MRI. J Head Trauma Rehabil (2007) 22(3):141–55. 10.1097/01.HTR.0000271115.29954.27 17510590

[109] Kranowitz C Preschool Sense. Preschool Sensory Scan for Educators. Las Vegas: Sensory Resources (2005).

[110] Mutti M Martin N Sterling H Spalding N Quick Neurological Screening Test. 3rd ed USA: Academic Therapy Publications (2012).

[111] Richter E Montgomery P The Sensorimotor Performance Analysis (SPA). Hugo, MN: PDP Press Inc (1995).

[112] Reisman J Hanschu B Sensory Integration Inventory Revised for Individuals with Developmental Disabilities (SII-R). USA: PDP Press (1999).

[113] Carmona S Hoekzema E Castellanos FX García-García D Lage-Castellanos A Van Dijk KR Sensation-to-cognition cortical streams in attention-deficit/hyperactivity disorder. Hum Brain Mapp (2015) 36(7):2544–57. 10.1002/hbm.22790 25821110 PMC4484811

[114] Scherder EJ Rommelse NN Bröring T Faraone SV Sergeant JA Somatosensory functioning and experienced pain in ADHD-families: a pilot study. Eur J Paediatr Neurol (2008) 12(6):461–9. 10.1016/j.ejpn.2007.11.004 18262449

[115] Parush S Sohmer H Steinberg A Kaitz M Somatosensory function in boys with ADHD and tactile defensiveness. Physiol Behav (2007) 90(4):553–8. 10.1016/j.physbeh.2006.11.004 17198716

[116] Martinez-Sanchis S The role of the prefrontal cortex in the sensory problems of children with autism spectrum disorder and its involvement in social aspects. Rev Neurol (2015) 60(S01):S19–24. 25726818

[117] Davies PL Gavin WJ Validating the diagnosis of sensory processing disorders using EEG technology. Am J Occup Ther (2007) 61(2):176–89. 10.5014/ajot.61.2.176 17436840

[118] Brett-Green BA Miller LJ Gavin WJ Davies PL Multisensory integration in children: a preliminary ERP study. Brain Res (2008) 1242:283–90. 10.1016/j.brainres.2008.03.090 18495092 PMC6390285

