# Corrigendum: Assessment of Sensory Processing Characteristics in Children between 3 and 11 Years Old: A Systematic Review

**DOI:** 10.3389/fped.2017.00266

**Published:** 2017-12-12

**Authors:** Sara Jorquera-Cabrera, Dulce María Romero-Ayuso, Gemma Rodriguez-Gil, José-Matías Triviño-Juárez

**Affiliations:** ^1^Centro de Terapia Infantil AYTONA, Madrid, Spain; ^2^Facultad de Terapia Ocupacional, Logopedia y Enfermería, Psychology, Universidad de Castilla-La Mancha, Talavera de la Reina, Spain; ^3^Facultad de Ciencias de la Salud, Departamento de Fisioterapia, Universidad de Granada, Granada, Spain; ^4^West Health District, Primary Care Center Francia, Madrid Health Service, Madrid, Spain

**Keywords:** assessment, children, sensory integration, sensorial modulation, sensory processing

**Error in Figure/Table**

In the original article, there was a mistake in Table 2 “Tools selected for the assessment of sensory processing in children aged 3 to 11 years”. We want to clarify that the errors we have corrected in this document occurred when transcribing the data. We want to clarify that these errors have not been intentioned

**Table 2:** Tools selected for the assessment of sensory processing in children aged 3–11 years.

Tool	Objective	Population	Applicability	Psychometric properties	Language in which the tests are available and the psychometric scores
Sensory processing measure (SPM) (52)	To assess sensory processing, praxis and social participation in different school environments and at home	SPM (5–12 years): home form, main classroom form, and school environments form. SPM-P (2–5 years) home and school forms	The scale is completed by teachers and caregivers who have known the child for more than a month	SPM was standardized with a sample of 1,051 typically developing children from the USA and Canada, aged 5–13 years. Also, 345 children receiving occupational therapy treatment was used to verify that SPM could help us differentiate typical children from those with clinical disorders. SPM-P was standardized with 651 typically developing children from the USA aged 2–5 years. Also, a sample of 242 children with occupational therapy treatment was used to verify that SPM-P let us differentiate typical children from those with clinical disorders. Good reliability and validity. Internal consistency (alpha coefficient) ≥0.75 for all scales and forms. SPM scales appropriately distinguished between a normative sample and a sample of clinic-referred children with sensory processing difficulties	Sensory Processing Measure-Hong Kong Chinese version (SPM-HKC). Cronbach’s alpha 0.80. ICC of the Main classroom form ranged from 0.82 to 0.98 and the ICC of the home form ranged from 0.70 to 0.95. Good discriminant validity. Moderate correlation between Sensory profile Chinese and SPM-HKC. It is available in Danish, Finnish, Norwegian, Swedish, and Arabic
Sensory profile (1,45,54)	Evaluates the type of responses and self-regulation strategies used by the child and the type of neurological threshold for different sensory stimuli	Different versions. It can be administered from 0 to 14 years. There is a second version (SP^2^) toddler, infant, child, short form (SSP) and school companion published in 2014	Scale is completed by teachers and parents	Sensory Profile was standardized with a sample of 1,037 children without disabilities, 32 children with autism and 61 with ADHD diagnosis. New version of Sensory profile, Sensory Profile 2 was standardized with a sample of 1,376 school-age children in the USA	Infant/toddler sensory profile. ICC > 0.90. Alpha coefficients varied from 0.40 to 0.74. Test–retest reliability = 0.81–0.90. India Sensory Profile Caregivers Questionnaire The interrater reliability (ICC = 0.87), test–retest reliability (ICC = 0.90), internal consistency (Cronbach’s α = 0.86), section total correlation, face, and content validity for the SPCQ were good. A threshold score of ≤481 in SPCQ was considered ideal as a cutoff score to identify cases of sensory processing dysfunction among Indian children. Sensory Profile for Chinese children with a good internal consistency (Cronbach’s α = 0.82). Test–retest reliability over a 2-week period (*r* = 0.93)
				ICC = 0.80–0.90 good test–retest reliability across quadrants, for factors ICC = 0.69–0.88 years ICC = 0.50–0.87 for scores in the composites of sensory processing, modulation, and behavioral and emotional responses. Internal consistency of the sections ranges from 0.70 to 0.90	
				SSP has a discriminant validity of >95% in identifying children with and without sensory processing differences	
Sensory Integration and Praxis Test (SIPT) (6)	To assess children’s sensory integration and praxis problems	Children aged from 4 to 8 years 11 months	Comprises 17 tests. Administered using visual demonstration and spoken instructions, except when assessing praxis. The lower the score, the greater the difficulty	Standardized with a sample of 1997 children in the USA. High psychometric properties	Available only in English, for USA population
DeGangi-Berk Test of Sensory Integration (TSI) (58)	Conducts a screening of SI dysfunction, with emphasis on the vestibular system. Assessment of postural and components and praxis. It is based on Assessment of Sensorimotor Integration in Preschool Children (DeGangi, 1979) (66)	Infant population aged 3–5 years	Comprises 36 items and assesses posture control, bilateral motor integration and reflex integration. The child completes various tests. Administration time is 30 min	Validity of domain and construct, stable inter-observer 0.84 and test–retest reliability. Standardized with a sample of 101 typical children and 38 developmental delayed children from US population	Available only in English
Touch Inventory for elementary school-aged children (TIE) (61)	Measures tactile defensiveness	Population 6–12 years. The criteria for administration are that the child needs to have the language competence of at least a 6-year-old, an IQ of at least 80 and no presence of physical disabilities (Royeen and Fortune 1990)	The 26-item Questionnaire. The response format for the TIE is 1 = no, 2 = a little, and 3 = a lot. Administered in 15 min, self-reported by child. The higher the score, the more the self-reported behaviors are indicative of tactile defensiveness	Standarized with a sample of 415 children from USA. Test –retest reliability (*r* = 0.91) with 1-week testing interval	Available only in English
Sensorimotor clinical observations (63–66)	Provides information on vestibular and proprioceptive functions. Mainly used to diagnose motor planning problems, vestibular, proprioceptive, proprioceptive-vestibular and motor deficits	From age 5	A tool that requires training and practice to be correctly administered and interpreted. Comprises 15 tests. Administration time between 30 and 40 min	High interrater reliability. Discriminative validity measured with a sample of children in Chile and the USA *p* < 0.01. Portuguese transcultural adaptation study (*N* = 201)	Available in English and Spanish
Comprehensive Observations of Proprioception (COP) (67)	The COP provides a reliable measure for detecting the origin of proprioceptive problems affecting children’s functional performance	Infant population from 2 years of age	Takes 15 min to administer and is designed for use in conjunction with sensorimotor observations or while observing a child’s free play	Sample size was 130 children. Intraclass correlation coefficient was 0.91. Validity found between results of COP and items from the SPM (body awareness) and the KIN (kinesthesia) and SWB (Standing and Walking Balance) tests from the SIPT	Available in English and Spanish
The Miller Assessment for Preschoolers (MAP) (68)	Assesses a child’s attention, social interaction, and sensory reactivity during the testing procedure provides a profile of sensory discrimination abilities, postural foundations, and praxis and screens for visual, perceptual, and language delays that could be affecting participation in the classroom	Test for children from 2 years, 9 months to 5 years, 8 months of age	Administration time 30 min. There are two forms: MAP Screening 27 Core test items (evaluation of attention, social interaction and sensory reactivity) and MAP Extended (behavior during testing, supplemental observations, developmental history: speech language, movement, draw a person), development history. 27 subtests in 5 domains: neurological foundations, motorcoordination, language, nonverbal cognition, and complex tasks (combined domains). The total MAP score is expressed in percentiles, and the cut-points are 0% to 5% (Red; likely problem, refer for evaluation), 6% to 25% (Yellow; possible problem, watch carefully and use clinical judgment about the need to refer for evaluation), and 26% to 99% (Green; unlikely to have problems, do not refer for assessment)	The MAP was standardized with a sample of 1,014 children. The MAP has excellent internal reliability (r = 0.79–0.82) and interrater reliability (r = 0.98). Test–retest reliability for total score is r = 0.81 Content validity for the MAP is supported in the literature as MAP total score correlates significantly with the WISC-R IQ scale (*r* = 0.50–0.45) and with the Woodcock-Johnson Math, Reading and Language subtests (*r* = 0.38–0.35)	Available in English, Japanese and Hebrew
Sensory Experiences Questionnaire Version 3.0 (SEQ-3.0) (7,69–72)	To obtain sensory characteristics and discriminate sensory patterns of hypo- and hyper-responsiveness among persons with autism, mental or developmental retardation	For 2–12 years	It is a 105-item parent report tool designed specifically to measure behavioral responses to naturally occurring sensory stimuli in the context of everyday situations in children with ASD. SEQ measures the frequency of sensory behaviors across four sensory response patterns (hypo-responsiveness, hyper-responsiveness, sensory interests, repetitions and seeking behaviors and enhanced perception), five modality categories (i.e., auditory, visual, tactile, gustatory/olfactory, vestibular/proprioceptive), and two contexts (i.e., social and non-social). The first 97 items measure the frequency using a 5-point Likert scale ranging from 1 (*never/almost never*) to 5 (*always/almost always*) with a higher score indicating more sensory symptoms. Caregiver takes approximately 15–20 min to complete the questionnaire	Has good internal consistency and test–retest reliability. Useful for assessing children with ASD. Psychometric study was conducted with 358 caregivers	Available only in English
The Sensory Processing Scales (SPS) Version 2.0 (28)	Evaluates sensory reactivity in seven domains: tactile (self-care and materials), auditory (sounds and places), visual, olfactory, gustatory, and vestibular-proprioception	4–19	Consists of a performance assessment of different activities and a caregiver-report inventory and a self-report form for adults. The results propose classifications of sensory over responsivity, sensory under responsivity, and sensory seeking. Administered in approximately 1 h. Consists of 27 subtests and 72 items across seven sensory domains (visual, auditory, tactile, vestibular, proprioceptive, gustatory, and olfactory). The activities are designed to resemble sensory experiences in daily life that generate atypical behavioral responses in children with sensory problems. Items within each subtest are scored to reflect the person’s responses at three time periods: (1) during the activity, (2) after the activity (<15 s), and (3) during the transition to the next activity	Standarized sample of 128 participants. Internal consistency is moderate to high, interrater reliability is moderate, and internal validity is statistically significant. Overall internal consistency yielded a 0.94, and domain reliabilities ranged from 0.79 to 0.93 (internal reliability >0.4) and discriminant validity (*p* < 0.01). The SPS Assessment appears to be a reliable and valid measure of sensory modulation (scale reliability >0.90; discrimination between group effect sizes >1.00). This scale has the potential to aid in differential diagnosis of sensory modulation issues	English
Test of Ideational Praxis (TIP) (73)	To examine a child’s ability to recognize and to interact with an and to evaluate ideation as a component of praxis	From 5 to 8 years. There is also a version for preschoolers, elaborated in 2014	A child is given a 24-inch long shoelace and is given the instruction, “Show me everything you can do with this string” and is then given 5 min to demonstrate the actions. A point is given for each action but the action must be demonstrated; description alone is not enough	Studies conducted in 2014 with 78 children aged 3, 4, and 5 years found, after 2 weeks, that the TIP had a high interrater reliability of 0.94 and a good test–retest reliability of 0.80	English
Motor Planning Maze Assessment (MPMA) (73)	To be used as a screening tool to identify deficits in motor performance and planning aspect of dyspraxia	Preschoolers from 3 to 5 years	Individually administered test consisting of three mazes. Application and correction takes 5 min	Has only been administered to 80 children in the USA. Interrater reliability was excellent on the total MPMA score [interclass correlation coefficient (0.96) and individual maze scores (0.90–0.98)]. The total MPMA score can distinguish developmental differences among preschoolers ages 3, 4, and 5 years. No differences were observed according to gender, race, or educational approach	English
Pediatric Clinical Test of Sensory Interaction for Balance (CTSIB) (74)	To evaluate a child’s ability to use visual, somatosensory, and vestibular input to maintain balance while standing	Over 6 years of age	The child must complete six tests, three on a stable surface and three on an unstable one. Some of the tests are performed with eyes closed and others with eyes open. In all conditions, the objective is to maintain balance for at least 30 s. Administration time is approximately 20 min	A tool with excellent interrater reliability (*r* = 0.88, range 0.60–1.00) for children between 4 and 9 years old. The sample data was 24 typical children. Validity of criteria: with proprioceptive disorders and the SOT. CTSIB shows which children have more modulation disorders and more reduced postural control than typically developing children for all visual stimuli (*p* < 0.05), except for somatosensory input with vision. There are only data from studies conducted in the USA. There is also a version for adults and older children	English
Classroom Sensory Environment Assessment (CSEA) (75)	Promote therapist–teacher collaboration to provide student support and classroom modification, for research on the impact of the sensory environment for children with ASD	Elementary school aged	161 items divided into sections by sensory type: vision (47), hearing (50), touch (20), movement (vestibular and proprioceptive; 25), smell (15), and taste (4). Items for the cafeteria, recess, and playground were included. The teachers rated items on the basis of a typical week. Teachers rated the frequency of occurrence of the sensory experience as no, never, or not applicable; rarely; occasionally; sometimes; and always. Next, if applicable, the teachers rated the intensity of the experience as weak, moderate, or strong	Classroom data (*N* = 152) were analyzed with counts, frequencies, means, and SDs. Reliability was examined with internal consistency ratings using Cronbach’s alpha. Skew and kurtosis were examined using the Kolmogorov–Smirnov test of normality and histogram. Interrater reliability was analyzed with intraclass correlation coefficients. The tool’s internal consistency is acceptable. Interrater reliability values did not reach acceptable levels in the pilot using the teacher–therapist rating pairs and total score. The ICC was −0.197. Cronbach’s alpha = 0.94. The current phase (Phase 4) included collection of descriptive data from a variety of elementary classrooms using the current version of the CSEA and an initial investigation of its internal consistency	English
Preschool Imitation and Praxis Scale (PIPS) (77,78)	The purpose of the Preschool Imitation and Praxis Scale (PIPS) is designated to be a reliable and valid multidimensional instrument to measure the accuracy of imitation performance of preschool children	1.5–4.9 years	40 PIPS items and 10 task categories of the PIPS: six gestural, three procedural and one facial. The positive and strong associations between the PIPS scale score and scores on mental, language and motor measures in children with autism spectrum disorders supported criterion-related validity	Psychometric study was conducted with 119 typically developing children. They demonstrated acceptable intra- and interrater reliability at the item level (0.45–1.00) and scale level. Exploratory factor analysis disclosed four dimensions on the scale: goal directed versus non-goal directed, procedural imitation, and single versus sequential bodily imitation. Internal consistency for the PIPS scale (*a* = 0.97) and subscales was high (a ranged from 0.79 to 0.96). In both samples, the PIPS scale score was strongly related to age (*r* = 0.78, respectively, *r* = 0.56). Significant relationships between the PIPS score and mental, language, motor ages in the ASD sample supported criterion-related validity (*r* ranged from 0.59 to 0.74)	English

The corrected Table 2 appears below. The authors apologize for this error and state that this does not change the scientific conclusions of the article in any way.

The original article has been updated.

## Conflict of Interest Statement

The authors declare that the research was conducted in the absence of any commercial or financial relationships that could be construed as a potential conflict of interest.

