# Supplementary material for: Assessment of Sensory Processing Characteristics in Children between 3 and 11 Years Old: A Systematic Review
Source: Front Pediatr. 2017 Mar 30;5:57. doi: 10.3389/fped.2017.00057 (PMC5371598; doi:10.3389/fped.2017.00057)
Supplement: Supplementary file 1 [file Data_Sheet_1.DOCX]

*Annex 1.* Table of articles excluded and reasons

| ***AUTHORS / YEAR*** | ***TITLE*** | ***REASON FOR EXCLUSION*** |
| --- | --- | --- |
| McFall, SA,  Deitz, JC,  Crowe, TK. (1993) | Test-Retest Reliability of the Test of Visual Perceptual Skills with Children Learning Disabilities | The publication date is prior to the inclusion criteria in the systematic review, and it focuses fundamentally on visual perception components. |
| Jirikowic, TL,  Engel, JM,  Deitz, JC. (1997) | The Test of Sensory Functions in Infants: Test-Retest Reliability for Infants with Developmental Delays | The publication date is prior to the inclusion criteria in the systematic review. |
| May-Benson TA  Koomar JA. (2010) | Systematic Review of the Research Evidence Examining the Effectiveness of Interventions Using a Sensory Integrative Approach for Children | It is a systematic review on the efficacy on sensory integration intervention and not on assessment. |
| Koening & Rudney (2015) | Performance Challenges for Children and Adolescents with Difficulty Processing and Integrating Sensory Information: A Systematic Review | It was published after the search period but was excluded as it focuses on the results of sensory problems and not on their assessment. |
| Lang R,  O’Reilly M,  Healy O,  Rispoli M,  Lyndon H,  Streusand W,  Davis T,  Kang S,  Sigafoos J,  Lancioni G,  Didden R,  Giesbers S. (2012) | Sensory integration therapy for autism spectrum disorders: A systematic review | The systematic review focuses on the efficacy of sensory processing intervention and not on assessment. It specifically studies autism spectrum disorder. |
| Mailloux Z,  May-Benson TA,  Summers CA,  Miller LJ,  Brett-Green B  Burke JP,  Cohn ES,  Koomar JA,  Parham LD,  Roley SS,  Schaaf RC,  Schoen SA. (2007) | Goal Attainment Scaling as a Measure of Meaningful Outcomes for Children With Sensory Integration Disorders | It presents a tool for evaluating the achievement of objectives and the results of treatment. It does not focus on assessment of sensory processing in infants. |
| May-Benson TA,  Roley SS,  Mailloux Z,Parham LD,  Koomar J,  Schaaf RC,  Jaarsveld AV,  Cohn E. (2014) | Interrater Reliability and Discriminative Validity of the  Structural Elements of the Ayres Sensory Integration Fidelity Measure | This work focuses on the criteria for monitoring the extent to which an intervention is implemented as conceptualized in the Ayres approach, not on the assessment of sensory processing in infants. |
| Roley, SS,  Mailloux, Z,  Parham, LD,  Schaaf, RC,  Lane, CJ,  Cermak, S. (2015) | Sensory Integration and Praxis Patterns in Children with Autism | It was published after the search period but was excluded as it focuses on sensory problems in children with autism and not with the assessment of these problems. |
